# MitoSuite: a graphical tool for human mitochondrial genome profiling in massive parallel sequencing

**DOI:** 10.7717/peerj.3406

**Published:** 2017-05-30

**Authors:** Koji Ishiya, Shintaroh Ueda

**Affiliations:** Department of Biological Sciences, Graduate School of Science, The University of Tokyo, Tokyo, Japan

**Keywords:** MitoSuite, Mitochondrial genome, Next-generation sequencing, Graphical tool, Massively parallel sequencing

## Abstract

Recent rapid advances in high-throughput, next-generation sequencing (NGS) technologies have promoted mitochondrial genome studies in the fields of human evolution, medical genetics, and forensic casework. However, scientists unfamiliar with computer programming often find it difficult to handle the massive volumes of data that are generated by NGS. To address this limitation, we developed MitoSuite, a user-friendly graphical tool for analysis of data from high-throughput sequencing of the human mitochondrial genome. MitoSuite generates a visual report on NGS data with simple mouse operations. Moreover, it analyzes high-coverage sequencing data but runs on a stand-alone computer, without the need for file upload. Therefore, MitoSuite offers outstanding usability for handling massive NGS data, and is ideal for evolutionary, clinical, and forensic studies on the human mitochondrial genome variations. It is freely available for download from the website https://mitosuite.com.

## Introduction

The human mitochondrial (mt) genome encodes important information that governs the development of various diseases (Taylor & Turnbull, 2005). It also reflects maternal lineage (Mishmar et al., 2003; Macaulay et al., 2005; Behar et al., 2008) and evolutionary history (Cann, Stoneking & Wilson, 1987; Torroni et al., 2006; Underhill & Kivisild, 2007). High-throughput, next-generation sequencing (NGS) technologies allow more rapid sequencing of a larger number of samples than does traditional capillary sequencing based on Sanger, Nicklen & Coulson (1977) method. NGS technologies also allow whole genome sequencing, exon sequencing, and gene expression profiling at high speeds and low costs (Metzker, 2010). The advent of these high-throughput technologies has led to a dramatic improvement in studies on the human mitochondrial genome. For instance, NGS has aided the discovery of variants and heteroplasmic mutation in the human mitochondrial genome (Tang & Huang, 2010). In addition, NGS data can help estimate the probability of exogenous DNA sources in forensic samples (Just, Irwin & Parson, 2015).

Consequently, the demand for advanced tools for analyzing the massive volume of data that NGS generates has also increased. Currently, there are several command-line tools available to analyze high-throughput sequencing data for the mitochondrial genome. MitoSeek (Guo et al., 2013) is one such character-based tool that provides information on mtDNA copy numbers, and alignment quality, somatic annotations, and structural variants of the mitochondrial genome. MToolBox (Calabrese et al., 2014) is another bioinformatics pipeline for analyzing mitochondrial genome data from NGS platforms, with functions similar to those of MitoSeek. There are also several web-based tools to analyze such data. For instance, MitoBamAnnotator (Zhidkov et al., 2011) assesses the functional potential of heteroplasmy. mit-o-matic (Vellarikkal et al., 2015) is another web-based pipeline for clinical annotations of mtDNA variants. However, these tools have some limitations with regard to uploading files on their servers. For example, the maximum file size that can be uploaded to mit-o-matic is restricted to less than 25 MB. To address this issue, in this study, we developed MitoSuite, a stand-alone tool which does not involve file-uploading procedures like web-based tools. The “uploading-free” process offers advantage for shortening actual run time, since it eliminates file-uploading and queue times required to begin analysis. Furthermore, the uploading-free platform is suitable for leakage prevention of personal genome data in clinical or forensic cases. MitoSuite also provides a graphical user interface (GUI), which offers user-friendly operability for researchers who are unfamiliar with the command-line interface. Our tool comprehensively supports quality check of alignment data, variant annotation, building consensus sequences, haplogroup classification, and detection of heteroplasmic sites, exogenous contamination, and base-substitution patterns for mitochondrial genome data obtained by high-throughput sequencing. The output summary is provided in the HTML format, which can be easily visualized using a web browser, without complicated programming processes. To our knowledge, MitoSuite is the first standalone, GUI software for comprehensive profiling of the mitochondrial genome, using high-throughput sequencing data with intuitive operability.

## Material and Methods

### Format for input data

MitoSuite supports the BAM format, a binary version of Sequence Alignment/Map (SAM), which is a tab-delimited text format for high-throughput sequencing alignment (Li et al., 2009). Since the genome size of mitochondria is small (approximately 17 kb), it is easy to manipulate the mitochondrial genome in simple text files such as those in FASTA format. However, because FASTA files do not contain information on either sequencing quality or alignment processes, it is difficult to detect problems with base-call errors or contamination, using sequence data in the FASTA format. In contrast, BAM files contain alignment conditions or base substitutions at each position of the mitochondrial genome, as well as reads of high-throughput sequencing. By using BAM files, MitoSuite can not only check mapping and sequencing quality, but can also detect mismatches potentially attributed to exogenous contamination, sequencing errors, or heteroplasmy. The input file is mtDNA alignment data, a BAM file mapped against a reference sequence of the human mitochondrial genome. MitoSuite supports multiple human mitochondrial reference sequences, including not only rCRS (Andrews et al., 1999), but also RSRS (Behar et al., 2012), chrM in hg19, chrMT in GRCh37, and chrMT in GRCh38.

### Test datasets

We used seven sets of empirical sequencing data (NA11920, HG01112, NA18941, HG00096, HG00273, NA18548, NA18510) of 1000 genomes project data (1000 Genomes Project Consortium, 2012) to evaluate the performance of MitoSuite for high-coverage sequencing data, as well as empirical ancient sequencing data of an ancient hunter-gatherer (Olalde et al., 2014) to examine whether this tool can detect ancient DNA profiles. These empirical data (BAM file) were converted to FastQ files by the SamToFastQ command in Picard tools (http://broadinstitute.github.io/picard), and then realigned to the human mitochondrial reference sequence rCRS, using the Burrows-Wheeler Aligner (BWA) (Li & Durbin, 2009). After the realignments, duplicated reads were removed from the BAM files by the MarkDuplicates command in Picard tools. Sequence reads for the ancient hunter-gatherer were aligned against rCRS, and duplicated reads were removed in the same way. Next, to check the accuracy of mitochondrial haplogroup assignment, we generated simulated NGS reads using 324 worldwide mitochondrial genome sequences (Table S1) with ART, a simulation tool to generate synthetic NGS reads (Huang et al., 2012). These sequence sets were selected from PhyloTree (Van Oven & Kayser, 2009) (http://www.phylotree.org), and included all known macro-haplogroups in nearly equal proportions. We obtained GenBank accession numbers (https://www.ncbi.nlm.nih.gov/genbank/) from the sub tree pages on PhyloTree’s site (e.g.,  http://www.phylotree.org/tree/L0.htm), and then downloaded FASTA files from GenBank, based on the accession numbers obtained with our in-house Python scripts. Based on the Illumina sequencer model in ART (ver.03.19.2015), we assumed 1% sequencing error, single-end 100-base reads, and average depth of 1–1000× in the simulated data. We aligned these simulated reads against rCRS using BWA, and then used these BAM files as simulation datasets.

### File processing

**Figure 1:**
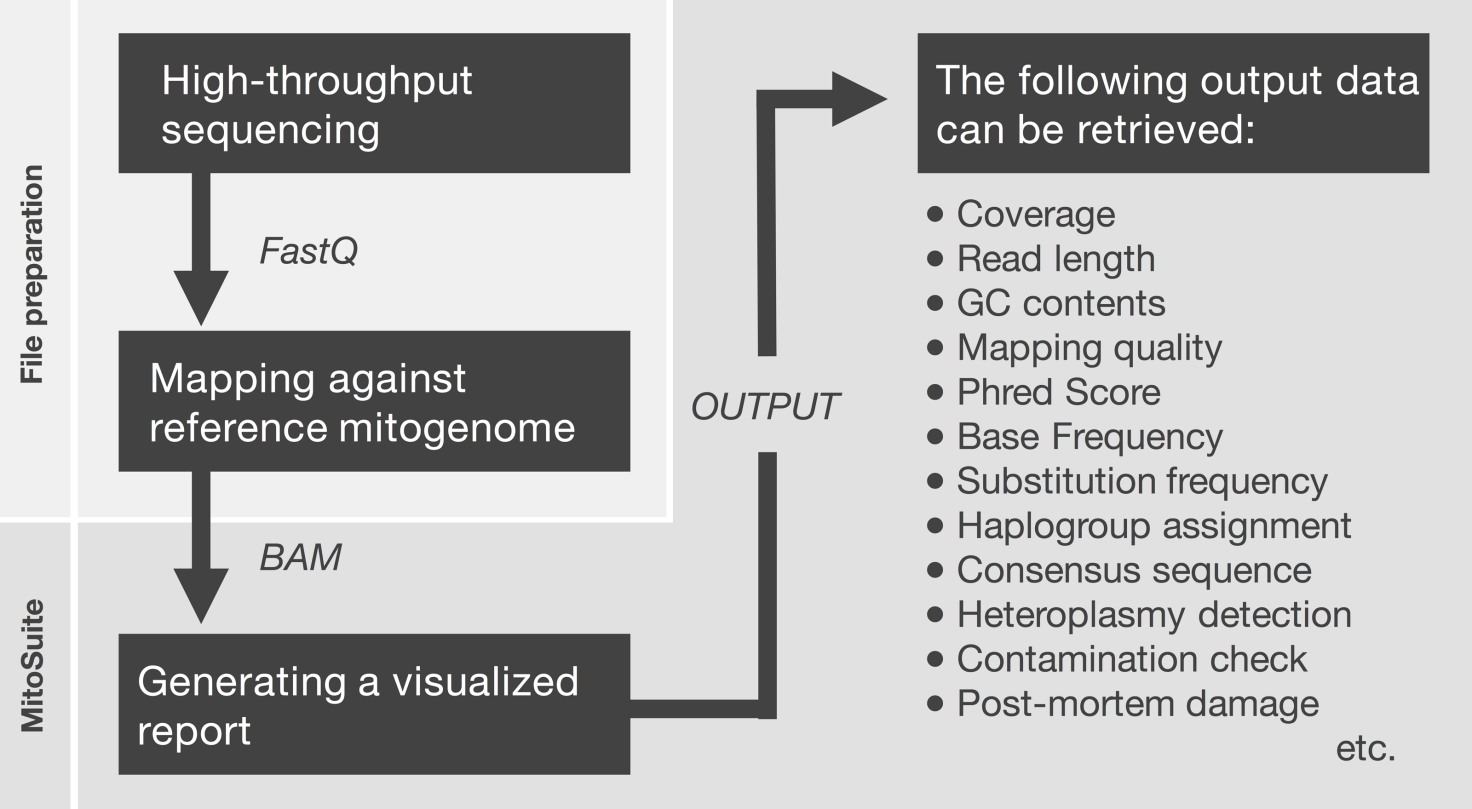
File preparation and processing flow for MitoSuite. BAM, binary version of SequenceAlignment/Map (SAM) format; FastQ, the format storing sequences and base call qualities. FastQ files are mapped against the mitochondrial genome by a mapper tool (e.g., Burrows-Wheeler Aligner). After file preparation, the BAM file is used as an input file for MitoSuite. Post-mortem damage means a series of DNA damage that occurs after biological death. These damages are observed as base substitution of C to T (G to A) and fragmentation of the DNA sequence (Briggs et al., 2007). Phred score is a quality value when a sequencer calls bases. This value is defined as phred score = −10 log 10 *P*_error_, where *P*_error_ is the probability of a sequencing error.

First, MitoSuite parses a BAM file and extracts reads together with the alignment condition involved with file headers, read groups, and reference sequences. Next, this tool automatically calculates summary statistics, including the depth of coverage, GC-content, base-call quality, mapping quality, and read length. These are important statistics for the quality control of NGS data. Moreover, this tool directly estimates mitochondrial haplogroups from a BAM file. MitoSuite does not require file format conversion (e.g., BAM > FASTA, BAM > VCF) and can directly assign mitochondrial haplogroups, based on the haplogroup-defining sites of PhyloTree. Figure 1 shows the schema for the file processing that can seamlessly work as an all-in-one tool.

### Detection of heteroplasmic sites

MitoSuite can also detect heteroplasmic sites that may be attributed to exogenous contamination, sequencing errors, amplification errors, or heteroplasmy. Our tool outputs a list of heteroplasmic positions with frequency greater than the minor allele frequency (MAF), which represents the frequency of inconsistent bases with the consensus sequence. MAF, a threshold for the detection of heteroplasmic sites, is given as follows:

\begin{eqnarray*}\mathrm{MAF}={N}_{\mathrm{diff}}/{N}_{\mathrm{con}} \end{eqnarray*}

where *N*_diff_ and *N*_con_ are the number of bases different from and identical to the consensus sequence, respectively. This means that MAF can be used as a threshold for the detection of heteroplasmic sites. MitoSuite also verifies the mitochondrial genome assembly by calculating the percentage of supporting bases of the consensus sequences in a BAM file (Fig. S1A). This percentage (*P*_support_) is computed as follows:

\begin{eqnarray*}{P}_{\mathrm{support}}=({N}_{\mathrm{agree}}/{N}_{\mathrm{depth}})\times 100 \end{eqnarray*}

where *N*_agree_ is the number of bases concordant with the assembled consensus sequence at each site, and *N*_depth_ is the depth of coverage at each site. This percentage provides clues to find unexpected contaminated sites (Fig. S1B).

### Optional functions

MitoSuite also provides a few optional functions to meet user needs for other data profiles. These functions can be accessed by selecting the relevant option menus. The optional ‘Annotation of disease-related variants’ function provides an annotation list of disease-associated mutations, based on the list of reported mitochondrial DNA base substitution diseases at MITOMAP (Kogelnik et al., 1996) (http://www.mitomap.org/MITOMAP). To accommodate private and local genetic data in medical and forensic cases, MitoSuite also supports customizable polymorphic databases. Customizable annotation information is required to correspond to the position of rCRS. The annotation file is a common comma-delimited CSV format containing two items: a mutation allele with a genomic position corresponding to that of rCRS (e.g., C150T, A4282G), and related information (e.g., related-disease name) in each designated column. The template of the annotation file is available from MitoSuite’s support page or can be downloaded by the installer. In this option, MUSCLE (Edgar, 2004) program is used to realign a consensus sequence against a reference sequence because MitoSuite finally takes the positional consistency of the obtained consensus sequence against the reference sequence (rCRS). Our tool can also calculate the percentage of each base substitution relative to the reference sequence in the total mapped reads, and then provide a pie chart showing the proportion of each base substitution. This chart will help users find locally biased substitution patterns in total mapped reads. Biased substitution patterns are often caused by the sample or experimental conditions, rather than by the natural process of mutations. For instance, deamination of cytosine to uracil, a postmortem hydrolytic change, often occurs in ancient DNA (Briggs et al., 2007). With the optional ‘Ancient DNA checker’ function, MitoSuite can detect postmortem damages and calculate the percentage of bases inconsistent with the haplogroup-defining variants to estimate exogenous contamination. This percentage (*P*_mismatch_) is computed as follows:

\begin{eqnarray*}{P}_{\mathrm{mismatch}}= \left[ \sum (i=1\rightarrow k)\{({N}_{\mathrm{mismatch}}/{N}_{\mathrm{match}})\}/k \right] \times 100 \end{eqnarray*}

where *k* is the total number of haplogroup-defining sites, *N*_mismatch_ is the number of bases inconsistent with the defining variant, and *N*_match_ is the number of bases consistent with the defining variant.

### Software availability

MitoSuite is freely available from https://mitosuite.com. Our tool mainly supports UNIX-like operating systems (OS) such as Mac OSX and Linux. The tool can also run on Linux-like environments (e.g., Cygwin) for Windows OS. MitoSuite for Mac OSX also provides the graphical installer package. This package can perform automatic installation without any command-line operations by the user. Installation instructions, tutorial movies, and additional technical support for MitoSuite are provided at https://mitosuite.com.

## Results and Discussion

**Figure 2:**
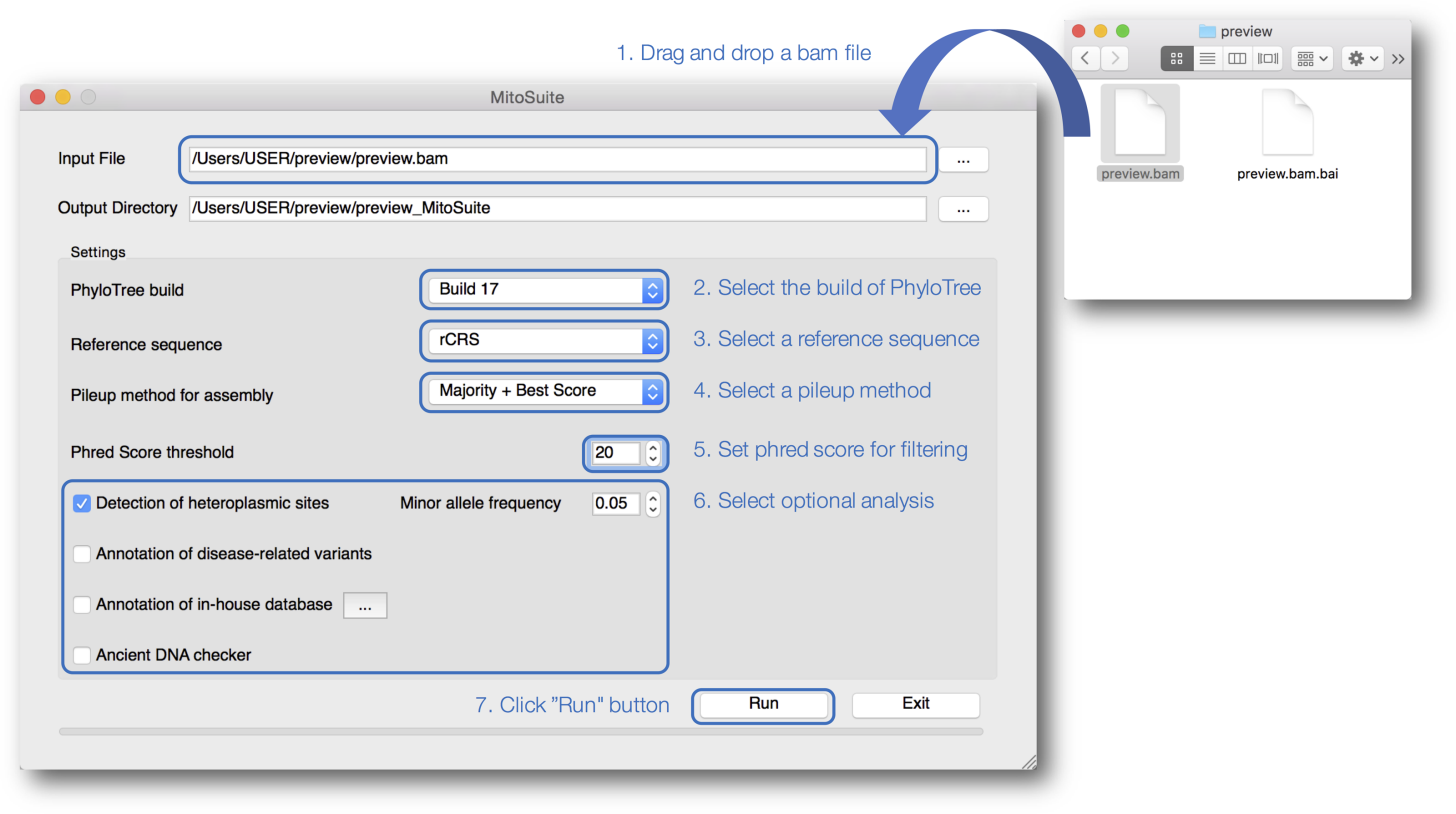
A screenshot of the graphical user interface of MitoSuite. At the beginning of analysis, users can drop and drag the input file (.bam) and click “Run” after setting the optional parameters (“Detection of heteroplasmic sites” shown selected). Bullet points 1–7 point out the protocol step-by-step.

**Figure 3:**
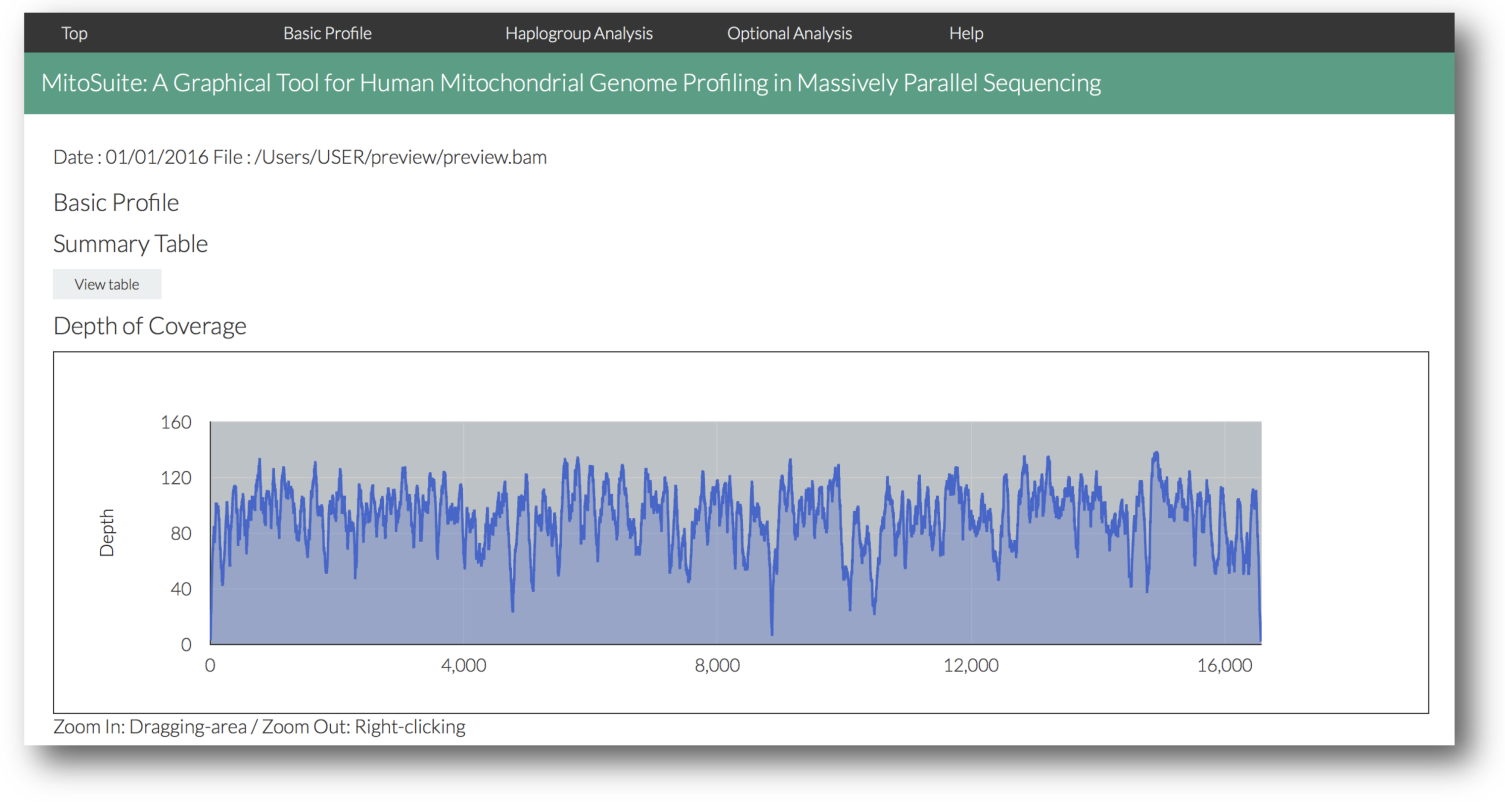
A screenshot of the visualized outputs of MitoSuite. The top page in an output file (results.html) is shown. After completion of analysis, users can quickly access the detailed information by clicking the link menu in the output page.

Our tool is designed for better usability, especially for non-bioinformaticians unfamiliar with typing complicated commands (Fig. 2). MitoSuite provides a drag-and-drop functionality for loading a BAM file and automatically displays an output destination directory. Our tool supports the latest build 17 and the previous build 16 of PhyloTree for the haplogroup assignment, and five available human mitochondrial reference sequences (rCRS, RSRS, hg19, GRCh37, and 38). MitoSuite also provides three options (Majority, Best Score, and Majority + Best Score) for calling a consensus base at each site. The “Majority” option decides the base by the majority in counting based rules. Thus, under this option, the most-read base at the site is adopted as a consensus base. For example, when counting only bases with a phred score higher than 30 defined as a base-call quality value at a site (when the base call threshold is set to 30), where the read depth of base “A” is 8 and that of base “T” is 2, the base “A” is adopted as a consensus base at a site. To avoid calling uncertain bases as much as possible, MitoSuite adopts “N” as the consensus base when multiple bases have the same read depth (e.g., A = 8, T = 8). The “Best Score” option decides the base with the highest basecall quality (phred score) at each site. The phred score is defined as the quality value when a sequencer calls bases. This option determines a consensus base at a site, using only the value of the Phred Score, regardless of the read depth. For example, when considering only bases with a phred score higher than 30 at a site, where the highest phred score of base “A” is 31, that of base “T” is 33, that of base “G” is 30, and that of “C” is 30, then the base “T” is adopted as the consensus base at the site even if base “T” is read less frequently than the other bases. Since this option does not take the read depth into consideration, it can also be applied to sites where bases cannot be determined by the majority option. However, this option adopts “N” as a consensus base at a site when multiple bases have the same maximum phred score. The “Majority + Best Score” option incorporates the “Best Score” with the “Majority” option at each site. This option firstly decides a base at each site by placing priority on majority rule, and then remaining sites that are not decided under majority rule are called by the “Best Score” option. For example, even if base “A” and base “T” have the same read depth at a site, base “A” will be adopted as a consensus base if it has the highest phred score. It is also necessary to set a threshold value for the phred score because MitoSuite adopts only bases with a phred score greater than the threshold values set by users to performs mtGenome assembly as well as haplogroup assignment, and detection of heteroplasmic sites. A consensus sequence is built on based on these basecall conditions and outputted as a FASTA file. MitoSuite also provides optional functions for detection of heteroplasmic sites, disease association, and ancient DNA according to their own purposes. The results of these analyses are finally outputted as a single html file (Fig. 3).

MitoSuite outputs analytical results for the quality of alignment data and genetic profiles that are haplogroup and polymorphisms on the mitochondrial genome (Fig. 3, Figs. S2–S4). As the results are provided in HTML format, they can be easily viewed on a web browser without depending on specific computer environments. The main output items are as follows: (1) A summary statistics table, including categories on data quality and genetic profiles (Fig. S2); this table shows an overview of the NGS data. Interactive dynamic charts for the mitochondrial genome operate with zoom and pan functions, which are useful for users to view the depth of the NGS data across mitochondrial genome (Fig. S3). (2) Figure S4 shows the other output data. All the data are saved in their respective output folders, and it is possible to individually access them. The distribution of read length, GC-content, and mapping quality are provided as histograms. Further, “retrieval” and “sort” functions in the data tables allow access to each item. These tables can be used for a quick check of quality as well as mutations at a desired destination site. MitoSuite can also automatically build a consensus sequence in the FASTA format from a BAM file, from which the phylogenetics or population genetics of the sequence can then be easily analyzed.

MitoSuite can be run even for deep sequencing data in a stand-alone environment. Here, we used seven high-throughput sequencing data from the 1000 Genomes Project as test data sets. Some of the datasets surpassed 1000× depth of coverage, and our tool was easily able to analyze these ultra-deep data. Analysis of a sample dataset with MitoSuite is shown in Table 1. It takes about 1 min to analyze 1000× ultra-deep data at the default settings, on a desktop computer equipped with a 3.5-GHz processor and 16-GB RAM. The time required is mainly for read operation, since the tool works in a stand-alone environment. MitoSuite also successfully detected the fragmentation and deamination pattern of ancient DNA-like on empirical reads from Olalde et al. (2014) (Figs. S4C, S4E). In addition, the most likely haplogroup estimated by our tool is “U5b2c1” that is consistent with reported one in Olalde et al. (2014) (Table 1).

**Table 1:** Sample dataset analyzed with MitoSuite.

Sample	Age	Haplogroup	Depth (avg)	Run (min)	Reference
NA11920	Modern	H1a1a1	1,516	1.5	1000 Genomes Project
HG01112	Modern	A2ac1	1,504	1.4	1000 Genomes Project
NA18941	Modern	N9b1a	1,297	1.2	1000 Genomes Project
HG00096	Modern	H16a1	1,212	1.1	1000 Genomes Project
HG00273	Modern	U5b1b2a	1,117	1.1	1000 Genomes Project
NA18548	Modern	C4a1b	1,021	1.1	1000 Genomes Project
NA18510	Modern	L0a1a3	746	0.8	1000 Genomes Project
La Brãna1	7,000 BP	U5b2c1	93	0.2	Olalde et al. (2014)

**Notes.**

avg average

**Figure 4:**
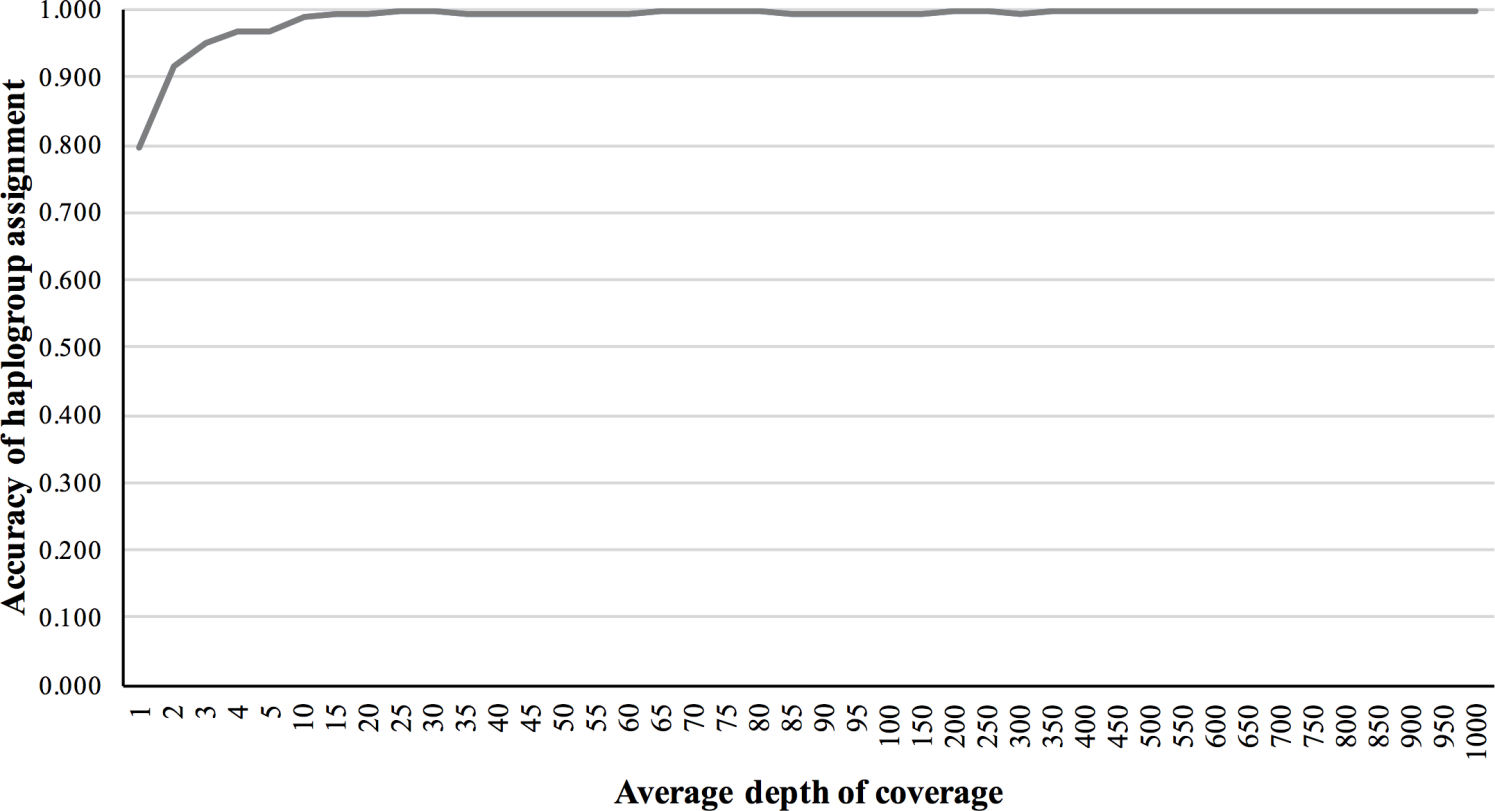
Haplogroup assignment accuracy of MitoSuite for simulated NGS reads generated from 324 worldwide mitochondrial genome sequences.

We also validated the accuracy of mitochondrial haplogroup assignment, using simulated NGS reads including data from all macro-haplogroups. The accuracy of haplogroup assignment is computed as follows: TP/(TP + FP). True positive (TP) is the number of haplogroups predicted and validated. False positive (FP) is the number of haplogroups predicted but failed in validation. The haplogroup assignment accuracy for 5×, 10×, 50×, 150×, and 200× fold coverage sets were 0.969, 0.991, 0.994, 0.994 and 0.997, respectively, assuming 1% base-call error (Fig. 4).

To examine whether our tool can detect heteroplasmic sites previously reported in empirical high-throughput sequencing data, we used MPS raw read data from Avital et al. (2012). We set MAF > 10% as the detection threshold after performing quality control analyses, including trimming of duplicates and low-quality bases (Phred Score < 20). Consequently, MitoSuite detected 14 out of 15 heteroplasmic sites with MAF > 10% in Avital et al. (2012) (Table S2). We think that the differences in quality control procedures and mapping tools between Avital et al. (2012) and this study may have changed heteroplasmic fraction in the alignment data.

**Table 2:** The list of available bioinformatics tools for mtDNA analysis.

	MitoSuite	HaploGrep2	mtDNA-Server	mtDNAprofiler	MToolBOX	MitoSeek
Input file	BAM	FASTA, hsd, VCF	FastQ, BAM, VCF	FASTA, variant^a^	FastQ, BAM, SAM	BAM
User interface	GUI	Web	Web	Web	CUI	CUI
Supported reference sequence	rCRS, RSRS, hg19^c^, GRCh^c^	rCRS, RSRS	rCRS, RSRS	rCRS	rCRS, RSRS	rCRS, hg19^c^
Automatic installation	✓	–	–	–	–	–
File upload	–	✓	✓	✓	–	–
Haplogroup assignment	✓	✓	✓	–	✓	–
mtGenome assembly	✓	–	✓	✓	✓	✓
Coverage plot	✓	–	✓	–	–	–
Quality check	✓	–	✓	–	–	✓
Concordance check	✓	–	–	✓	–	–
Damage check	✓	–	–	–	–	–
Contamination check	✓	–	✓	–	–	–
Relative copy number	–	–	–	–	–	✓
Variant annotation	✓	–	✓	–	✓	✓
Structural variants detection	–	–	–	–	–	✓
Somatic mutation detection	–	–	–	–	–	✓
Heteroplasmy detection	✓	–	✓	–	✓	✓

	MitoBamAnnotator	mtDNAmanager	MitoTool	HAPLOFIND	mit-o-matic	Phy-Mer
Input file	BAM	variant^a^	FASTA, variant^a^	FASTA	FastQ, pileup^b^	FASTA, FastQ, BAM
User interface	Web	Web	Web/GUI	Web	Web/CUI	CUI
Supported reference sequence	rCRS	rCRS	rCRS, RSRS	rCRS, RSRS	rCRS	reference- independent
Automatic installation	–	–	–	–	–	–
File upload	✓	✓	✓	✓	✓	–
Haplogroup assignment	–	✓	✓	✓	✓	✓
mtGenome assembly	✓	–	–	–	✓	✓
Coverage plot	–	–	–	–	–	–
Quality check	–	–	–	–	–	–
Concordance check	–	–	–	–	–	–
Damage check	–	–	–	–	–	–
Contamination check	–	–	–	–	–	–
Relative copy number	–	–	–	–	–	–
Variant annotation	✓	–	✓	✓	✓	–
Structural variants detection	–	–	–	–	–	–
Somatic mutation detection	–	–	–	–	–	–
Heteroplasmy detection	✓	–	–	–	✓	–

**Notes.**

✓ available – not-available

a A text-based format for describing SNPs information against a reference seuquence.

b A text-based format for describing the base-pair information of the reads against a reference sequence.

c Support for the mitochondrial sequence (chrM/chrMT) on hg19, GRCh37 and 38.

Useful bioinformatics tools for various mtDNA studies have been developed and are currently available to researchers. Mitochondrial haplogroup is an important genetic profile for molecular anthropological and forensic genetic investigations, and most available mtDNA tools support haplogroup assignment for various data formats. MitoTool (Fan & Yao, 2013), mtDNAmanager (Lee et al., 2008), and HAPLOFIND (Vianello et al., 2013) can estimate haplogroup, using the FASTA format or a text-based format containing variant information against a reference sequence. HaploGrep2 (Weissensteiner et al., 2016a) also supports the VCF format storing DNA polymorphism data, as well as the two above-mentioned formats. mtDNA-Server (Weissensteiner et al., 2016b), MToolBox, mit-o-matic, Phy-Mer (Navarro-Gomez et al., 2015), and MitoSuite can manipulate massively parallel sequencing (MPS) data such as the FASTQ or BAM (SAM) formats for haplogroup classification. MToolBox, MitoSeek, MitoBamAnnotator, mtDNA-Server, mit-o-matic, and MitoSuite can annotate variants in high-throughput sequencing data. The detection of heteroplasmy from a single individual or tissue provides useful information in clinical or forensic cases. MToolBox, MitoSeek, MitoBamAnnotator, mtDNA-Server, mito-o-matic, and MitoSuite can also report possible point heteroplasmy (PHP), based on detection parameters (e.g., minor allele frequency; MAF) set by users.

Users can select a suitable tool that takes into consideration their application, computational environment, data size, and bioinformatics skills. Command-line tools such as MitoSeek or MitoToolBox have the advantage of flexible incorporation into customizable NGS pipelines, but their setup is still difficult for non-bioinformaticians. MitoSeek is a useful tool for the detection of structural variants or somatic mutations, but its use requires installation of Circos, which is a software package for genomic data visualization (Krzywinski et al., 2009). This in turn requires users to install several dependent Perl modules based on their computational environment (e.g., operating system), as well as to set the local path to executable files. Command-line tools sometimes change their command specifications when updating the version. Therefore, users need to appropriately manage the version of dependent command-line tools for proper functioning of the pipeline and set an environment path in the local host, because the pipelines contain several command-line tools, including mapper or variant callers (e.g., BWA, Picard tools, GATK McKenna et al., 2010). Web-based tools such as mit-o-matic or mtDNA-Server do not require complicated installation processes, and provide a system that is easy to use. However, there are still unavoidable issues that include file size limits or queue times required to start analysis on the web-server. In addition, it is often necessary to assign an email address or individual account to manage uploaded data, and few servers clarify what technology is being used in the background of the management system. Users thus need to trust the server-side management system. Indeed, the mitochondrial genome is widely used in medical and forensic fields, and thus analysis environments must be very restrictive in terms of security systems. Our tool provides a server-independent, stand-alone system that brings advantages especially to medical and forensic researchers in terms of security. MitoSuite also provides a graphical user interface with intuitive operability, in addition to a graphical report on quality of alignment data, variant annotation, building of consensus sequences, haplogroup classification, detection of heteroplasmic sites and exogenous contamination, damage detection, and interactive dynamic graphics across the complete mitochondrial genome in NGS data (Table 2). MitoSuite for Mac OSX also provides an easy-to-use automated installer. Therefore, it provides a user-friendly solution for many investigators unfamiliar with advanced information-processing techniques. We expect our tool to promote human mitochondrial genome studies in the fields of anthropological science, forensic casework, and medicine.

##  Supplemental Information

10.7717/peerj.3406/supp-1 Supplemental Information 1 Supplementary Figures Figure S1. Support ratio in simulated reads. We used simulated reads of two distinct haplogroups (H and L0). These simulated reads are assumed 1% base-call error, average depth of 1–100 ×, and 0 –30 % contamination rate that is the percentage of the number of contaminated reads in the total reads. (A) Two figures are scatter plots output from MitoSuite in the presence (30%) and the absence (0%) of exogenous contaminants, respectively. (B) This figure shows the support ratio for all simulated data set (1-100 ×; 0-30%).
Figure S2. Summary statistics table.
Figure S3. Screenshot of the MitoSuite output area chart for depth of coverage across the human mitochondrial genome. Std; Standard deviation, Q1; 25th percentile, Q3; 75th percentile, Max; Maximum depth, Min; Minimum depth, >10-50; genome coverage rate over the depth value (10-50).
Figure S4. Different types of outputs given by MitoSuite. (A) Box plot of phred scores (base quality). (B) Line plot of base frequency at each position of reads. (C) Histograms of mapping quality (blue), read length (green), and GC-contents (yellow). (D) Pie chart of proportion of base substitutions. (E) Line plot of base substitutions at each position of reads. This plot shows the deamination of cytosine to uracil, which is a post-mortem hydrolytic change representative of ancient sequences.
Figure S5. Screenshot of annotation results. Upper table shows the annotation result of reported disease-related variants. Position column shows a reference sequence position (rCRS). Mutation column means that of Allele in MITOMAP (Jan. 04, 2017 version). Lower table shows the annotation result of an in-house customizable annotation database. The annotation file is a common comma-delimited CSV format containing two items: a mutation allele with a genomic position corresponding to that of rCRS (e.g., C150T), and related information (e.g., related-disease names) in each designated column. The template of the annotation file is available from MitoSuite’s support page (https://mitosuite.com) or can be download by the installer.

10.7717/peerj.3406/supp-2 Supplemental Information 2 Supplementary Tables

## Additional Information and Declarations

### Data Availability

The following information was supplied regarding data availability: We used seven sets of empirical sequencing data (NA11920, HG01112, NA18941, HG00096, HG00273, NA18548, NA18510) of 1000 Genomes Project data (1000 Genomes Project Consortium, 2012).
