# Supplementary material for: MitoSuite: a graphical tool for human mitochondrial genome profiling in massive parallel sequencing
Source: PeerJ. 2017 May 30;5:e3406. doi: 10.7717/peerj.3406 (PMC5452969; doi:10.7717/peerj.3406)
Supplement: Supplemental Information 1 — Figure S1. Support ratio in simulated reads. We used simulated reads of two distinct haplogroups (H and L0). These simulated reads are assumed 1% base-call error, average depth of 1–100 ×, and 0 –30 % contamination rate that is the percentage of the number of contaminated reads in the total reads. (A) Two figures are scatter plots output from MitoSuite in the presence (30%) and the absence (0%) of exogenous contaminants, respectively. (B) This figure shows the support ratio for all simulated data set (1-100 ×; 0-30%). Figure S2. Summary statistics table. Figure S3. Screenshot of the MitoSuite output area chart for depth of coverage across the human mitochondrial genome. Std; Standard deviation, Q1; 25th percentile, Q3; 75th percentile, Max; Maximum depth, Min; Minimum depth, >10-50; genome coverage rate over the depth value (10-50). Figure S4. Different types of outputs given by MitoSuite. (A) Box plot of phred scores (base quality). (B) Line plot of base frequency at each position of reads. (C) Histograms of mapping quality (blue), read length (green), and GC-contents (yellow). (D) Pie chart of proportion of base substitutions. (E) Line plot of base substitutions at each position of reads. This plot shows the deamination of cytosine to uracil, which is a post-mortem hydrolytic change representative of ancient sequences. Figure S5. Screenshot of annotation results. Upper table shows the annotation result of reported disease-related variants. Position column shows a reference sequence position (rCRS). Mutation column means that of Allele in MITOMAP (Jan. 04, 2017 version). Lower table shows the annotation result of an in-house customizable annotation database. The annotation file is a common comma-delimited CSV format containing two items: a mutation allele with a genomic position corresponding to that of rCRS (e.g., C150T), and related information (e.g., related-disease names) in each designated column. The template of the annotation file is available from MitoSuite [file peerj-05-3406-s001.pdf]

Supplementary figure legends

Figure S1. Support ratio in simulated reads. We used simulated reads of two distinct haplogroups (H and L0). These simulated reads are assumed 1% base-call error, average depth of 1–100×, and 0 – 30 % contamination rate that is the percentage of the number of contaminated reads in the total reads. (A) Two figures are scatter plots output from MitoSuite in the presence (30%) and the absence (0%) of exogenous contaminants, respectively. (B) This figure shows the support ratio for all simulated data set (1-100×; 0-30 %).

Figure S2. Summary statistics table.

Figure S3. Screenshot of the MitoSuite output area chart for depth of coverage across the human mitochondrial genome. Std; Standard deviation, Q1; 25th percentile, Q3; 75th percentile, Max; Maximum depth, Min; Minimum depth, >10-50; genome coverage rate over the depth value (10-50).

Figure S4. Different types of outputs given by MitoSuite. (A) Box plot of phred scores (base quality). (B) Line plot of base frequency at each position of reads. (C) Histograms of mapping quality (blue), read length (green), and GC-contents (yellow). (D) Pie chart of proportion of base

substitutions. (E) Line plot of base substitutions at each position of reads. This plot shows the deamination of cytosine to uracil, which is a post-mortem hydrolytic change representative of ancient sequences.

Figure S5. Screenshot of annotation results. Upper table shows the annotation result of reported disease-related variants. Position column shows a reference sequence position (rCRS). Mutation column means that of Allele in MITOMAP (Jan. 04, 2017 version). Lower table shows the annotation result of an in-house customizable annotation database. The annotation file is a common comma-delimited CSV format containing two items: a mutation allele with a genomic position corresponding to that of rCRS (e.g., C150T), and related information (e.g., related-disease names) in each designated column. The template of the annotation file is available from MitoSuite's support page (https://mitosuite.com) or can be download by the installer.

Figure S1 Support ratio in simulated reads

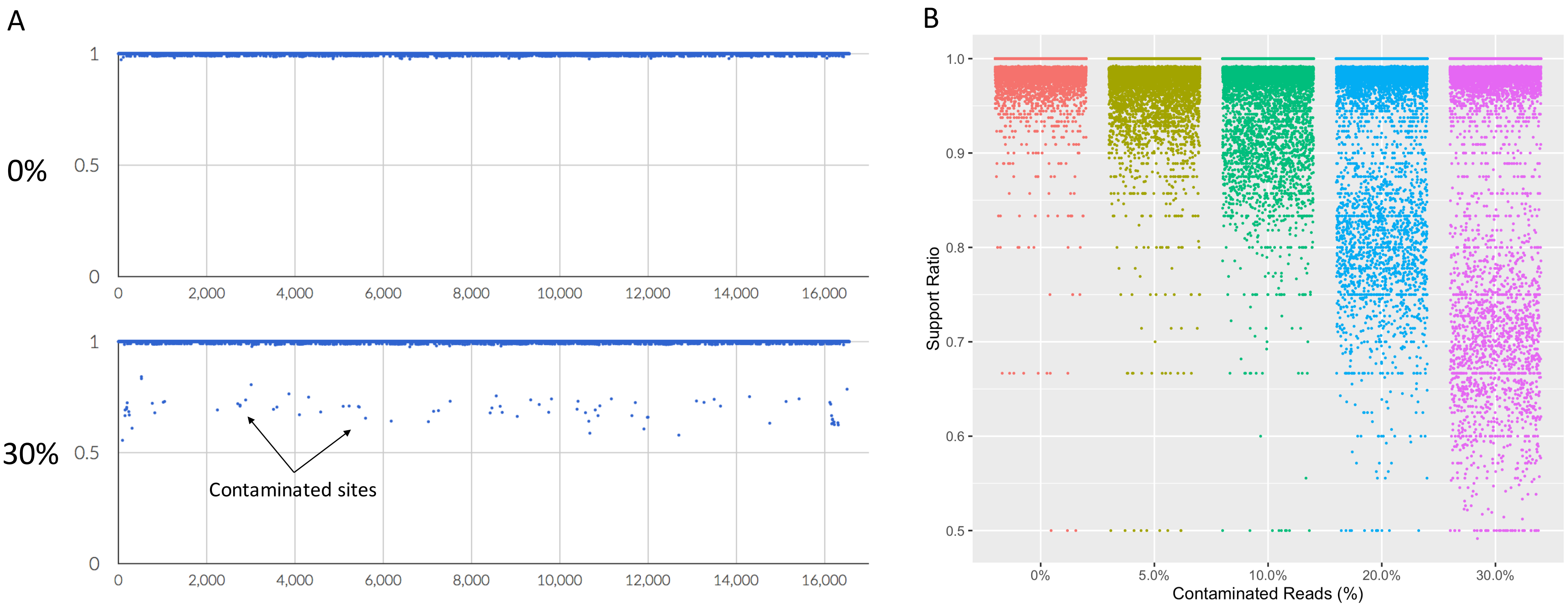

Figure S2 Summary statistics table

Summary Table

View table ← Click "View table" button to open the table below

Item	Value	Note
Total reads	21818	
Mapped reads	21818	Percentage of mapped : 100.0%
Unmapped reads	0	Percentage of unmapped : 0.0%
Duplicate reads	0	Percentage of duplicates : 0.0%
Reference name	gi|251831106|ref|NC_012920.1|	
Reference length	16569	
Phred encoding	Phred+33	
Read length (avg.)	70.333	
GC% (avg.)	44.575%	
MapQ (avg.)	36.821	
Depth of coverage (avg.)	92.619	
Phred score thereshold	20	
Mitogenome coverage (%)	100.0%	
Mapped sites (phred > 20)	16569	
Unmapped sites (phred > 20)	0	
Assigned haplogroup (phylotree build 16)	U5b2c1	the percentage of concordance of diagnostic sites : 100.0 %
Pileup method	Majority + Best score	
Assembled consensus sequence		
Observed base substitutions	6287	These are substitutions against an assembled consensus sequence.
The percentage of concordance of consensus bases (avg.)	99.3%	The percentage presents fraction of bases consistent with bases of an assembled consensus sequence.

Figure S3 Screenshot of the MitoSuite output area chart for depth of coverage across the human mitochondrial genome

Depth of Coverage

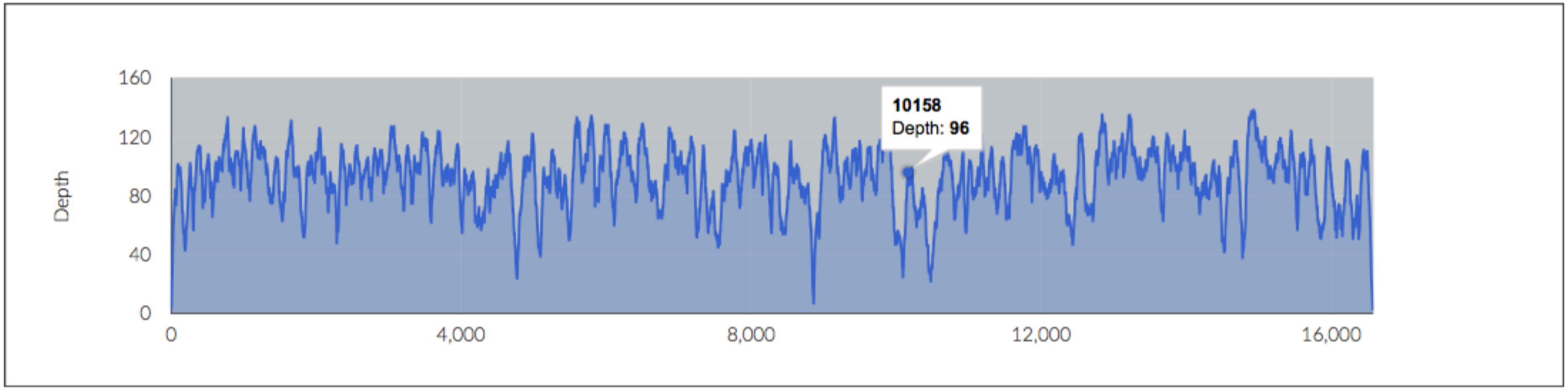

Average	Median	Std.	Q1	Q3	Max	Min	>10x	>20x	>30x	>40x	>50x
92.62	96.0	20.43	81.0	107.0	139.0	2.0	1.0	1.0	0.99	0.99	0.97

Depth table

Search

Position	Depth
1	3
2	5
3	5
4	5
5	5

Show rows: 5 Results: 1 - 5 of 16569

Std; Standard deviation, Q1; 25th percentile, Q3; 75th percentile, Max; Maximum depth, Min; Minimum depth, >10-50 ; genome coverage rate over the depth value (10-50).

Figure S4 Different types of outputs given by MitoSuite

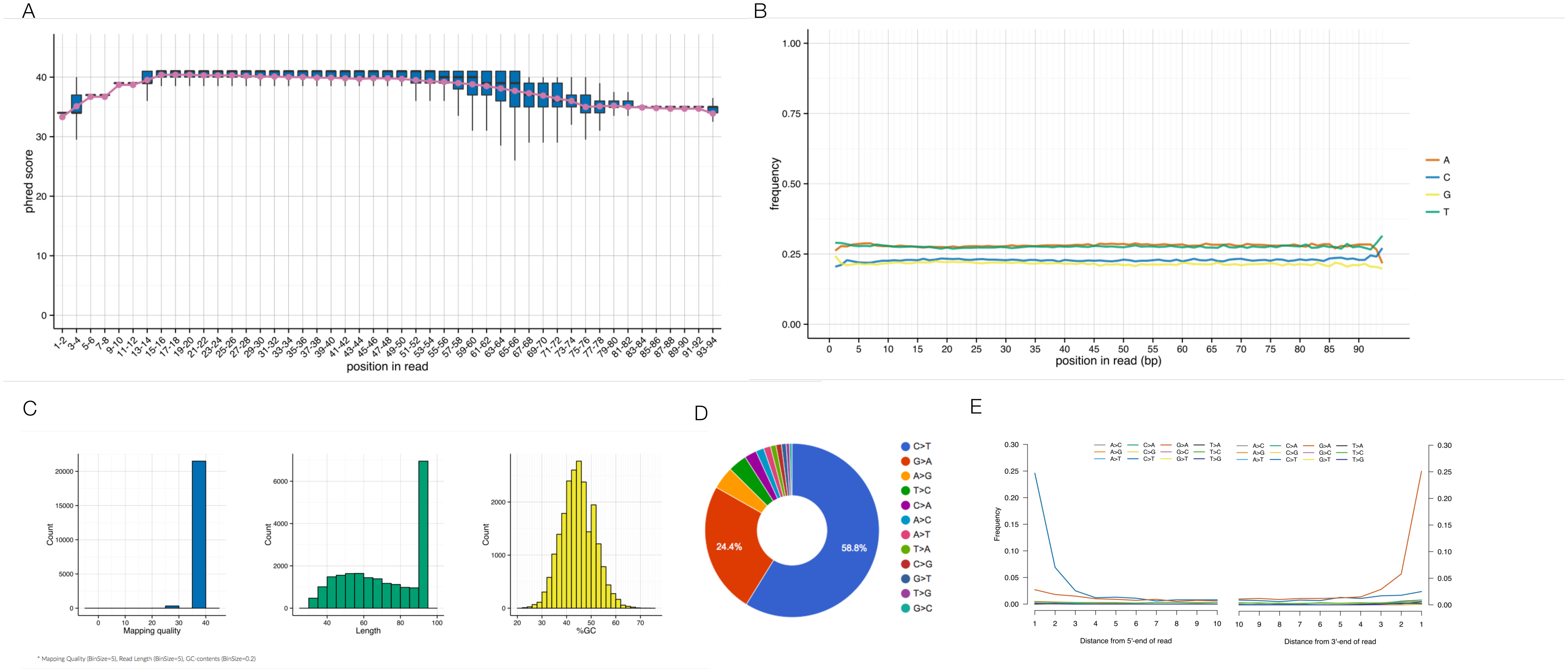

Figure S5 Screenshot of annotation results

Annotation of disease-related variants (β)

Reports of Disease-Associations in MITOMAP(Jan. 04, 2017 version).

Search

Position	Mutation	Reference	Query	Disease	MITOMAP References
150	C150T	C	T	Longevity / Cervical Carcinoma / HPV infection risk	C150T
4282	A4282G	A	G	CPEO Plus	A4282G
10398	A10398A	A	A	Invasive Breast Cancer risk factor; AD; PD; BD lithium response; Type 2 DM	A10398A
11467	A11467G	A	G	Altered brain pH / sCJD patients	A11467G
12308	A12308G	A	G	CPEO / Stroke / CM / Breast & Renal & Prostate Cancer Risk / Altered brain pH /sCJD	A12308G
12372	G12372A	G	A	Altered brain pH / sCJD patients	G12372A
13637	A13637G	A	G	Possible LHON factor	A13637G
16192	C16192T	C	T	Melanoma patients	C16192T
16270	C16270T	C	T	Melanoma patients	C16270T
16519	T16519T	T	T	Cyclic Vomiting Syndrome with Migraine /metastasis	T16519T

Show rows: 10 Results: 1 – 10 of 10

*Position column shows a reference sequence position (rCRS).Mutation column means that of Allele in MITOMAP.Please check more detail information at MITOMAP.

Annotation of an in-house database (β)

Mutations are annotated by using in-house_annotation_data.csv.

Search

Position	Mutation	Reference	Query	Annotation
114	C114C	C	C	annotation1
150	C150T	C	T	annotation2
195	T195T	T	T	annotation3

Show rows: 5 Results: 1 – 3 of 3

*Position column shows a reference sequence position (rCRS).
