# Supplementary material for: MitoSuite: a graphical tool for human mitochondrial genome profiling in massive parallel sequencing
Source: PeerJ. 2017 May 30;5:e3406. doi: 10.7717/peerj.3406 (PMC5452969; doi:10.7717/peerj.3406)
Supplement: Supplemental Information 2 [file peerj-05-3406-s002.pdf]

Table S1 Mitochondrial genome sequences to test haplogroup assignment in MitoSuite

Accession	Haplogroup (PhyloTree)
EU092714	L0a1a1
JX303911	L0a1a2
JQ044943	L0a1a3
EU092906	L0a4
HM771161	L0a2a1
EU092936	L0b
EU092870	L0f1
KC345899	L0k1a1
KC345791	L0d1a
KC345891	L0d2d
DQ112737	L1b1
JQ705275	L1c1a
AF346992	L1c2a1a
HM771222	L1c2b1a
EU273502	L1c4a
EU273489	L1c6
JX303797	L1c5
EU092703	L1c3a
EU273488	L1c3b1a
EU273493	L1c3c
DQ341060	L5a1a
EF556173	L5a1a
EU092888	L5a1b
EU092943	L5a1b
HM771198	L5a1c
EU092699	L5a2
DQ341061	L5b1a
EU092774	L5b1b
DQ304928	L2a1a
EU092761	L2a1b
EU092747	L2b1
EU092661	L2b3c
JQ044941	L2c
AY195785	L2c5
JQ045050	L2d
EU092794	L2d1a
EU092724	L2e

Accession	Haplogroup (PhyloTree)
FJ460523	L2e1a
EU092773	L6a
EU092802	L6a
DQ341063	L6b
EU092686	L6b
FJ460531	L4a1
DQ341064	L4a1a
EU092935	L4a2
JQ044811	L4b1a
EU092942	L4b2a1
EF184627	L4b2a2
EU092938	L4b2a2a
JQ702504	L4b2b1
JN655813	L3a1a
EU092726	L3b1a
EU092891	L3f1a
EU092660	L3c
DQ112884	L3d
EU092827	L3e1
DQ341069	L3i1b
EU092822	L3k
EF556171	L3x1a1
JN655830	L3h1a1
JQ702955	M1a
AP012349	M20
KC505097	M51a
DQ408676	M3
DQ408679	M4
AY922291	M65a1
EF556195	M5a
HM156679	M18a
JX289116	M38
HM346892	M9a1
AY519496	C1a
FJ951462	C4a1
FJ951515	C5a1
FJ951594	C7
FJ383641	C7b

Accession	Haplogroup (PhyloTree)
FJ951440	C5d1
FJ951614	C5c
FJ951472	C5b1
FJ951611	C4e
GQ895155	C4d
AY519493	Z1
FJ147318	Z1a
AP008426	Z2
AP008841	Z3
EU597518	Z3a1
FJ383644	Z3b
GU392051	Z4
AP013181	Z4a1a1
AP008553	Z5
FJ383629	Z7
GQ119027	E1a
EF093539	E1a1
EF185804	E1a1a1
HQ700849	E1a2
HQ700847	E1a2a
FJ428236	E1b
EF061150	E1b1
HQ700856	E2a
EF185816	E2b1
EF093542	E2b2
AP008845	G1a1a4
EF153773	G1a1
AY195762	G1b
AY255137	G1c
FJ198217	G2a1
FJ015040	G2b1a
JF824842	G2c
GU014566	G3a1
FJ748726	G3b1
AP008911	G4
DQ372885	Q1
AY289085	Q1a
AF347003	Q1b

Accession	Haplogroup (PhyloTree)
EU597543	Q1c
HQ113226	Q2a
GQ214525	Q2a1
EF495218	Q2b
AY289079	Q3a
AY289089	Q3a1
EF061146	Q3b
JQ704974	D4
JN253391	D1
AP011004	D4b2
AP008251	D4c1b
AP008628	D4d
AP008789	D4e1a
EU660536	D2a
JF824877	D2b1
AP008519	D5c1a
AP011006	D6a1
JQ245777	N1a1a
AY714031	N5
EU787451	N2a
KC867130	N3
KC887495	N7a1
JX289118	N8
AP008726	N9a1
EF495214	N13
DQ112753	N14
JF739542	N22
AP010699	A1
DQ282395	A2
AY963575	A6a
EF153771	A12
JF824996	A15a
EF153780	A16
AP013217	A3
AP008265	A5a
EU482363	A8
HM569228	A10
JQ245791	I

Accession	Haplogroup (PhyloTree)
HM454265	I1a
FJ968796	I1b
JQ705932	I1c
EU570217	I2
JQ702041	I3a
KJ021059	I4
JQ245724	I5
JQ705382	I6a
KF146253	I7
AY289059	O
DQ404447	O1
AY289056	O1a
AF346963	S1
JN226144	S1a
AY289051	S2
AY289066	S3
AY289062	S4
EF495220	S5
EU600318	X1a
EF177437	X3
GQ200588	X2
DQ523631	X2c
EU600325	X2h
JQ245730	X2i
FJ008043	X2m1
EF660942	X2n
JQ245731	X2o
HQ456226	X4
EU439939	X2a1
FJ825753	X2a2
EU935450	X2j
FJ457949	X2b
JQ705795	X2d
KF056262	W
JQ702793	W1
JQ705313	W3a
KF146279	W3b
KF146281	W4

Accession	Haplogroup (PhyloTree)
KF146285	W5
JQ245723	W6
HM352797	W7
JQ245778	W8
JQ245759	W9
EF153813	Y1
HM776715	Y1a
GU123044	Y1b
AY255138	Y1b1
JF824992	Y1b1a
AP008723	Y2
GQ119016	Y2a
GQ119013	Y2a1
KC994134	Y2a1a
AP008764	Y2b
KC985149	R1a
JQ705561	R1a1a
KC985159	R1a1
HM030522	R1a1b
KC985148	R1a1c
KC985165	R1b
JQ703633	R1b1
JX155266	R2a
EF556167	R2b
JX155271	R2c
AY255136	B6a
JX900371	B4a1a1
JQ704728	B2a
AY519494	B4b1a
AY255135	B4d1
KC733253	B4e
JN857017	B4j
KC521454	B4c1a
EU597566	B5a1a
JF824930	B5b1a
HM357817	F1a1a
AP010738	F1c
AP012346	F1f

Accession	Haplogroup (PhyloTree)
KF849909	F2
AY255167	F3a
AP013180	F3b
AP008744	F4a1a
AP013200	F4a1b
JF824892	F4a2
AY289095	F4b
JQ704041	J2a1
AY339579	J2a1a1a1
FJ348157	J2a1a1a2
JQ703568	J2a1a1a3
JQ705390	J2a1a1b
JQ797924	J2a2
EF660967	J2a2a
JQ797920	J2a2a1
JQ702563	J2b1
FJ213765	J2b2
AF347004	P1
DQ112897	P2
KC993994	P10
AY289052	P3a
EF061158	P4a
AY289063	P5
AY289053	P6
AY289054	P7
DQ404446	P8
KC993944	P9
KC405582	T1a
JQ798058	T2a1
AY495273	T2b
JQ798090	T2c
AY714037	T2d1a
EF177410	T2e
JQ703997	T2f
EU935442	T2g1
JN202724	T2h
JX462725	T3
JQ702678	R0a1

Accession	Haplogroup (PhyloTree)
HM185216	R0a1a
HM185266	R0a2
HM185261	R0a2b
HM185215	R0a2c
JF717359	R0a2d
HM185238	R0a2h
HM185265	R0a3
JQ702940	R0a4
JF717361	R0b
JQ705599	HV0
JF320654	HV1
AY713986	HV2
EF417833	HV4
EF419890	HV5
HQ658738	HV6
EU545443	HV7
EU545457	HV8
HM852849	HV12a
JF700125	HV13
HQ384174	H1
EU597492	H2a
AY738987	H3
EU935460	H4
AY495174	H5
HM765475	H6a
AY495120	H7
AY738957	H8a
EF660914	H31
HQ659700	H100
JQ702026	V1a
JQ705658	V2
JQ703666	V3
AY495312	V4
AY339451	V5
FJ348207	V6
JQ705798	V7
JQ702025	V8
EF177445	V9

**Accession**	**Haplogroup (PhyloTree)**
JQ704988	V23
HM852790	U1a1
KC521458	U5
EF064317	U6a
JQ702004	U2e2a4
FN600416	U2
HM852891	U3a
JQ703947	U4a1
AY882389	U9a
AY714004	U7
JQ702759	U8a
JQ706038	K1a
DQ301795	K1a1b1a
AY714044	K1a1b2a
JQ705454	K1a1c
JQ702168	K1b1a1a
AY495241	K2a
JX273249	K2b
JQ704064	K2b1b
DQ301796	K2c
HM852886	K3

Table S2 Detected heteroplasmic sites with MAF > 10 % in Avital et al., (2012).

Sample ID	mtDNA Position	Region	Cell Type	Heteroplasmic fraction (Avital et al.)	Heteroplasmic fraction (MitoSuite)	> 10% (Avital et al.)	> 10% (MitoSuite)	Run
95451	9077	ATP6	Blood	43.99	41.3	✓	✓	SRR409202
95451	9077	ATP7	Muscle	47.22	45.1	✓	✓	SRR420854
95452	9077	ATP7	Blood	38.53	48.9	✓	✓	SRR420827
95452	9077	ATP7	Muscle	41.82	28.7	✓	✓	SRR420855
70251	16213	D-LOOP	Blood	10.64	15.2	✓	✓	SRR420842
70251	16213	D-LOOP	Muscle	15.21	33.3	✓	✓	SRR420957
70252	16213	D-LOOP	Blood	11.81	12.2	✓	✓	SRR420844
68842	152	D-LOOP	Muscle	11.88	41.9	✓	✓	SRR420859
68842	11299	ND4	Muscle	11.52	11	✓	✓	SRR420859
68842	14167	ND6	Muscle	10.94	36	✓	✓	SRR420859
68842	15132	CYB	Muscle	48.71	18.9	✓	✓	SRR420859
68842	15132	CYB	Blood	48.11	39.5	✓	✓	SRR420831
68841	14167	CYB	Muscle	12.42	47.2	✓	✓	SRR420858
68841	15132	D-LOOP	Blood	41.96	21.3	✓	✓	SRR420830
51402	385	D-LOOP	Muscle	10.3	7.5	✓	-	SRR420863

Low-quality and duplicates reads were trimmed in QC procedures before we analyzed them using MitoSuite.
